# Inhibitory Effects of Apigenin on Tumor Carcinogenesis by Altering the Gut Microbiota

**DOI:** 10.1155/2020/7141970

**Published:** 2020-10-06

**Authors:** Shichang Bian, Hongjuan Wan, Xinyan Liao, Weisheng Wang

**Affiliations:** Tianjin Fourth Central Hospital, The Fourth Central Hospital Affiliated to Nankai University, The Fourth Central Clinical College, Tianjin Medical University, Tianjin, China

## Abstract

The flavonoid apigenin is common to many plants. Although the responsible mechanisms have yet to be elucidated, apigenin demonstrates tumor suppression *in vitro* and *in vivo*. This study uses an azoxymethane (AOM)/dextran sodium sulfate- (DSS-) induced colon cancer mouse model to investigate apigenin's potential mechanism of action exerted through its effects upon gut microbiota. The size and quantity of tumors were reduced significantly in the apigenin treatment group. Using 16S rRNA high-throughput sequencing of fecal samples, the composition of gut microbiota was significantly affected by apigenin. Further experiments in which gut microbiota were reduced and feces were transplanted provided further evidence of apigenin-modulated gut microbiota exerting antitumor effects. Apigenin was unable to reduce the number or size of tumors when gut microbiota were depleted. Moreover, tumor inhibition effects were initiated following the transplant of feces from mice treated with apigenin. Our findings suggest that the effect of apigenin on the composition of gut microbiota can suppress tumors.

## 1. Introduction

In 2012, it was estimated that there were 14.1 million new cancer cases worldwide, and by 2032, this figure is projected to increase to 25 million [1]. The incidence rate of malignant tumors was 199.4/10 million in 2012; 8.22 million deaths were attributed to malignant tumors, with a mortality rate of 116.3/10 million [1]. There is an urgent need to undertake more cancer prevention and treatment research to address this serious situation.

The number of symbiotic bacteria in the human gut is calculated to be approximately 100 trillion, weighing 1–2 kg. These symbionts are made up of more than 7,000 strains representing approximately 800 bacterial genera. The microbiota can be considered an immune organism contributing to the human host's health [2, 3]. The importance of gut flora to host health is highlighted by their critical functions of breaking down indigestible carbohydrates, inhibiting pathogenic bacterial infections, synthesizing vitamins, exerting antitumor effects, and modifying host immune reaction [4, 5]. Studies that compare the intestinal flora of tumor patients with that of healthy people reveal significant differences in the microbiota [6, 7]. Gut microbiota are an important factor in treating tumors [8].

Over the past 20 years, many researchers have focused on the anticancer properties of plant-derived polyphenols [9]. Polyphenolic compounds possess one or more aromatic rings and hydroxyl functional groups and are potential anticancer drug candidates [10]. Polyphenols are naturally occurring compounds present in fruit, nuts, vegetables, and plant-based products such as spices, tea, and wine [11]. There is considerable diversity among these plant secondary metabolites, with the compounds extending from simple small molecules to complex, highly polymeric compounds [12–14]. Natural polyphenols exhibit antioxidant and anti-inflammatory activities. They also have the capacity to influence molecular targets and signaling pathways, thereby modulating various processes, including angiogenesis, cell differentiation, migration, proliferation and survival, detoxification enzymes, hormone activities, and immune responses. It is these impressive qualities that are deemed responsible for the anticancer effects of natural polyphenols [15, 16].

Among other plant foods, the common flavonoid apigenin is found in oranges, onions, parsley, tea, and wheat sprouts [17]. The administration of 40–160 *μ*M of apigenin to H460 lung cancer cells resulted in damaged DNA and a concomitant increase in the production of reactive oxygen species (ROS) and Ca^2+^ [18]. Apoptosis was induced in gastric cancer cells by apigenin (20 *μ*g/mL); while normal gastric cells exhibited minimal cytotoxicity, undifferentiated gastric cancer cells were especially vulnerable [19]. Apigenin treatment (50 mg/kg) has been shown to be effective in inhibiting tumor growth and metastasis in the orthotopic colorectal cancer (CRC) model. At lower doses (20–120 *μ*M), cell proliferation, invasion, and migration have been reduced in a number of CRC cell lines [20].

The morbidity of CRC in rats fed a high-fat diet may be reduced by apigenin modulating gut microbiota; apigenin might also modulate the microbiota-gut-brain axis. The bacteria *Helicobacter pylori* responsible for stomach ulcers are also implicated in gastric cancer. Atrophic gastritis has been proposed as being a key precursor condition in stomach cancer induced by *Helicobacter pylori*. In a study of Mongolian gerbils, administering apigenin (30–60 mg/kg/week) successfully prevented the development of *Helicobacter pylori*-induced atrophic gastritis and subsequent gastric cancer [21]. The *in vivo* mechanisms responsible for the effects of apigenin have yet to be fully characterized; however, we hypothesize that the antitumor effects of apigenin are determined by its effect upon gut microbiota.

To explore the *in vivo* effects of apigenin on the composition of intestinal microbiota composition, carcinogenesis, and tumors in tumor-bearing mice, this study used high-throughput sequencing of 16S rRNA to refine the antitumor effects of apigenin on intestinal microbiota, and a fecal transplant experiment was conducted.

## 2. Materials and Methods

### 2.1. Animals

This research was approved by the animal use and welfare committee of Tianjin Fourth Central Hospital. All the experiments were performed according to the committee's animal welfare guidelines. Four-week-old female-specific pathogen-free (SPF) BALB/c mice were acquired from the Tianjin Laboratory Animal Center (Tianjin, China). Prior to experimentation, the mice were given 5 d to acclimatize to the laboratory environment. All animals were kept in an air-conditioned room (temperature 23°C ± 3°C; relative humidity, 50% ± 20%; ventilated fresh air > 17 times/h; 12 h light/dark cycle).

Dextran sulfate sodium (DSS) (MW 36–50 kDa) (Cat. No. 160110) was obtained from MP Biomedicals, LLC (Aurora, OH, USA). Mice were randomly allocated to two groups, a control group and an intervention (AP) group (*n* = 8 per group). Azoxymethane (Sigma-Aldrich) (10 mg/kg) was injected intraperitoneally to induce colon cancer. One week later, DSS (2.5%) was added to drinking water to last for 5 d, and then, plain drinking water was provided for the next 14 d. This 19 d cycle was repeated three times. The mice in the AP group received a 30 mg/kg apigenin supplement in their diet. All mice were sacrificed 4 weeks after DSS treatment via cervical dislocation.

Cephradine (cephalosporin) and gentamicin (aminoglycoside) broad-spectrum antibiotics were administered to deplete gut flora for the commensal microbe-depleted (CMD) experiment [22]. Cefradine was prepared at a concentration of 67 mg/kg, and gentamicin was prepared at a concentration of 2 mg/kg with a sterile saline solution. Antibiotics were administered by oral gavage b.i.d. for 30 d. To evaluate the efficiency of depletion, bacterial DNA was extracted. The mice were then randomly segregated into a CMD group and a CMD-AP group (*n* = 8 per group). The same protocol was applied to the groups treated with azoxymethane (AOM) and DSS. The diet of the mice in the CMD-AP group was supplemented with 30 mg/kg apigenin.

The surgical procedure for the fecal microbiota transplantation (FMT) experiment is as follows. Stools were collected from mice fed a 30 mg/kg apigenin-supplemented diet and normal SPF mice (control donor mice). The feces were collected daily under sterile conditions in laminar flow fume hoods, and the same group of feces was combined. Next, 100 mg of feces was resuspended in 1 mL sterile saline and then mixed using a benchtop vortex for 10 s. The solution was centrifuged at 800 g for 3 min, and the supernatant was collected to transplant microbiota. Fresh transplant microbiota was prepared 10 min before use to prevent changes in bacterial composition. The mice were separated into an FMT-AP group and an FMT control group (*n* = 8). The same AOM and DSS protocol that has been described previously was applied. The FMT control group mice received 0.1 mL transplant microbiota from control donor mice each day, and the FMT-AP mice received 0.1 mL transplant material from apigenin donor mice each day [23]. Throughout the experiment, each mouse was monitored individually.

### 2.2. Fecal Microbiological Determination by 16S rRNA Gene Sequencing

Prior to sacrifice, approximately 200 mg of fecal matter was collected from each rodent. Following the manufacturer's instructions, a commercial E.Z.N.A Stool DNA Kit (Omega Bio-Tek, Inc., Norcross, GA, USA) was used to isolate the higher-quality total microbial DNA. The 20 *μ*L reaction mixture for PCR comprised 10 ng template DNA, 1 *μ*L each of forward and reverse primers, 2 *μ*L dNTPs (2.5 mM), 4 *μ*L FastPfu buffer, and 0.5 *μ*L FastPfu polymerase; the mixture was supplemented with phosphate-buffered saline (PBS; Beyotime Biotech, Shanghai, China). The following PCR amplification process was applied: 95°C for 2 min, then 30 cycles at 94°C for 30 s, at 50°C for 30 s, at 72°C for 45 s, and at 72°C for 10 min. Using a DNA gel-extracting kit (Cat. No. AP-GX-250, Axygen Biosciences, Union City, CA, USA), the amplified PCR products were mixed and purified in accordance with the manufacturer's instructions. Using a DNA PCR-Free Sample Preparation Kit (Cat. No. FC-121-3003, Illumina, San Diego, CA, USA) combined with the pooled PCR products as templates, the gene library was built. To sequence the PCR products, equimolar amounts were pooled and then sequenced using the Illumina High-Throughput Sequencing Platform (mode: Hiseq2500; Illumina, San Diego, CA, USA).

### 2.3. Colon Inflammatory Index

Colons and tumors were harvested for histology. Samples were processed and fixed on slides before applying hematoxylin and eosin stains. The inflammatory index was analyzed according to a previous study [24]. The inflammatory index recorded the areas of epithelial degeneration and the severity, the presence of ulcers, epithelial erosions, tissue hyperplasia, the size of the affected area, and whether the areas were focal or multifocal.

### 2.4. Statistical Analysis

SPSS statistical software was used to analyze the results. To assess differences involving two groups, Student's *t*-test or one-way ANOVA was used. The data are expressed as mean ± standard deviation (SD), with *P* < 0.05 indicating statistical significance.

## 3. Results

### 3.1. Depressor Effect of Apigenin on Tumor Growth and Metastasis *In Vivo*

**Figure 1:**
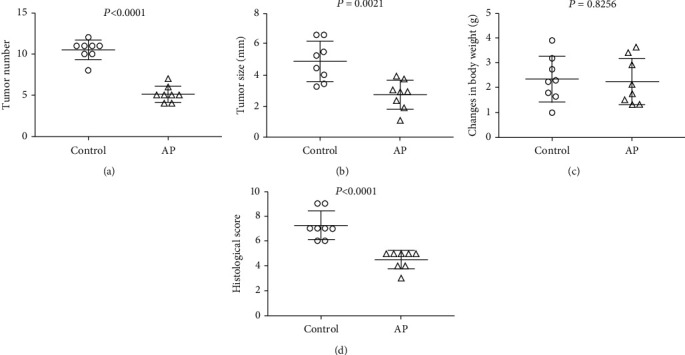
Apigenin decreased tumor morbidity in a CRC model related to colitis: the number (a) and size (b) of colon tumors in the control and AP groups, (c) change in body weight in the control and AP groups, and (d) histological score for the control and AP groups; *n* = 8.

A comparison was made of the weight and volume of tumors and the change in body weight between the AP mice and the controls (Figures 1(a)–1(c)). Although there were no changes in body weight, the control mice exhibited heavier and larger tumors (*P* < 0.05).  Figure 1(d) presents the histological scores, which are higher for the control mice (*P* < 0.05). This suggests that apigenin treatment might have helped the tumor-bearing mice to maintain some level of physical condition.

### 3.2. The Influence of Apigenin on Gut Microbiota in Tumor-Bearing Mice

**Figure 2:**
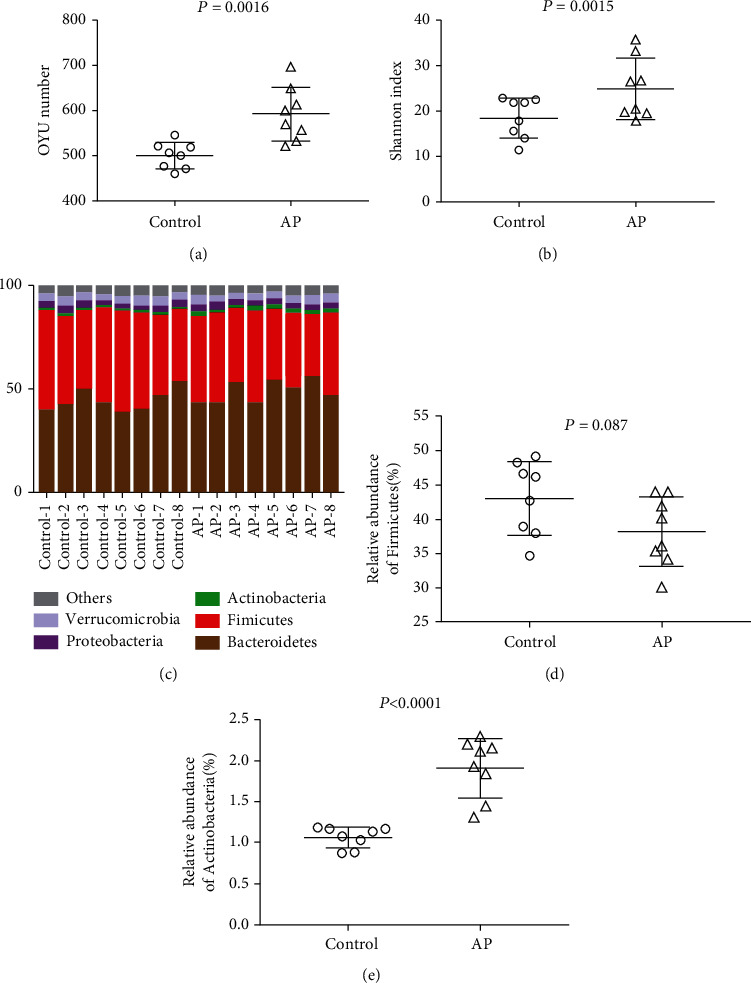
Results of 16S rRNA high-throughput sequencing: (a) OTU number and (b) Shannon index in the control (*n* = 8) and AP (*n* = 8) groups. (c) The heat map and the relative abundance of (d) Firmicutes and (e) Actinobacteria showing differences in the abundance of gut microbiota in the different groups at the phylum level.

To investigate apigenin-altered gut microbiota, the gut bacterial populations were determined using high-throughput sequencing. Figures 2(a) and 2(b) present the differences in the abundance of operational taxonomic units (OTUs) and the diversity index of the gut microbiota of the control and AP groups. Changes in the abundance of microbiota are illustrated in phylum-level heat maps of the community following the administration of apigenin (Figure 2(c)). There was a trend of decrease in Firmicutes (Figure 2(d)) and a significant increase in Actinobacteria (Figure 2(e)) in the AP group. Apigenin may exert antitumor effects that are responsible for changes to the phylum.

### 3.3. The Influence of Gut Microbiota on the Antitumor Effects of Apigenin

**Figure 3:**
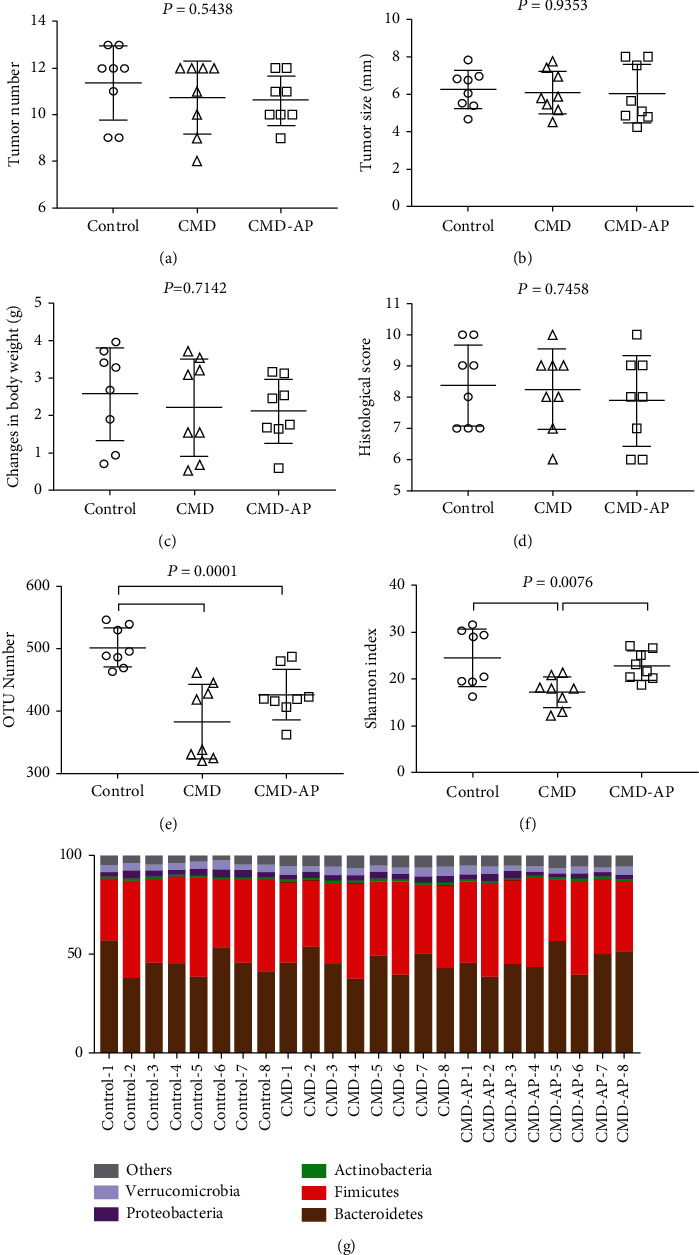
Influence of gut microbiota depletion on the antitumor effects of apigenin. The number (a) and size (b) of colon tumors in the CMD and CMD-AP groups; (c) change in body weight, (d) histological scores, (e) OTU number, (f) diversity indexes, and (g) the abundance of gut microbiota at the phylum level in the commensal microbe-depleted (CMD) experiment; *n* = 8.

To examine the effect of gut microbiota on the antitumor effects of apigenin, gut microbiota were reduced using cephaloridine and gentamicin. No significant difference in the antitumor role as determined by tumor size and volume was identified between the CMD group and the CMD-AP group (Figures 3(a) and 3(b)). This indicates that after depleting the gut microbiota, apigenin did not have any benefit on the general health of the mice (Figures 3(a) and 3(b)). Furthermore, there were no significant differences in the change in body weight or histological scores (Figures 3(c) and 3(d)). However, as Figures 3(d) and 3(e) depict, the amount of OTU and bacterial 1diversity was significantly decreased in the CMD groups relative to the controls. This confirms that the antibiotics had effectively depleted gut microbiota. The relative abundance of the microbiota is shown in Figure 3(g); we did not detect any microbial difference at the phylum level among the groups.

### 3.4. Regaining Antitumor Effects Using Fecal Microbiota from the Apigenin Treatment

**Figure 4:**
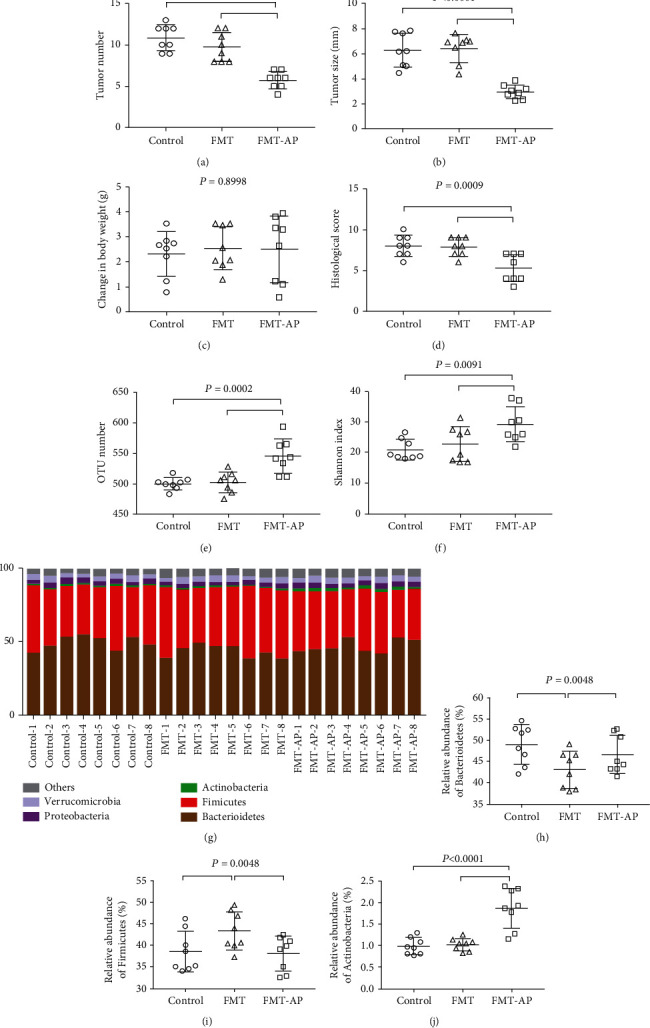
*In vivo* inhibition of tumor growth following fecal transplants. Number (a) and size (b) of colon tumors in the FMT (*n* = 8) and FMT-AP (*n* = 8) groups; (c) change in body weight, (d) histological scores, (e) OTU number, (f) diversity indexes, (g) the abundance of gut microbiota at the phylum level, and the relative abundance of (h) Bacteroidetes, (i) Firmicutes, and (j) Actinobacteria in the fecal microbiota transplantation (FMT) experiment; *n* = 8.

Freshly extracted supernatant from control donor and apigenin-fed (30 mg/kg) donor mice was intragastrically administered to the FMT model and FMT-AP groups over 14 d. The number and size of tumors in the FMT group of mice were significantly greater (*P* < 0.05) than in the FMT-AP model group (Figures 4(a) and 4(b)), whereas no difference in the change of body weight was detected (Figure 4(c)). Similarly, the histological score of the colon in the latter group was significantly lower (*P* < 0.05) (Figure 4(d)). Meanwhile, the amount of OTU and bacterial diversity was significantly increased in the FMT-AP group relative to the control and FMT groups (*P* < 0.05) (Figures 4(e) and 4(f)). The relative abundance of the microbiota is shown in Figure 4(g). The relative abundance of Bacteroidetes (Figure 4(h)) and Actinobacteria (Figure 4(j)) in the FMT group of mice was significantly lower (*P* < 0.05) than that in the FMT-AP model group, and the relative abundance of Firmicutes (Figure 4(i)) in the FMT group of mice was significantly greater (*P* < 0.05) than that in the FMT-AP model group.

## 4. Discussion

Each year, approximately 1.2 million people are diagnosed with CRC, which is the third most prevalent cancer. There is a high incidence of CRC in developed countries [25, 26]. There are approximately 1,000 different kinds of bacteria in the human body, amounting to 10^14^ individual bacteria. The sum of microbial genes is calculated to be at least 150 times more than the number of human host genes [27]. Gut microbiota are symbionts, contributing to host health and to the biological barrier that aids nutrient absorption, immune regulation, and energy metabolism [28]. Studies reveal that cancer patients present with gut microbiota disorders; therefore, the rationale is that by modifying gut flora, tumor-ameliorating effects may ensue. This study administered apigenin that moderated the antitumor effects of the microbiota.

Apigenin is one of the most bioactive flavonoids and is present in many fruits and vegetables [29]. Research into apigenin started in the 1960s; by the 1980s, it was postulated that the compound had cancer-preventing properties [29, 30]. The low intrinsic toxicity of apigenin has attracted the interest of many researchers [31]. Different types of cancer and normal cell types respond differently to apigenin, influencing cancer cell growth, survival, and apoptosis [29, 30]. Although apigenin is degraded by gut microbiota, this polyphenol and its metabolites may in turn modulate the structure and function of the gut microbiota. As yet, these modulatory effects on the microbiota remain largely uncharacterized [32].

Even though there has been considerable progress in understanding cancer over the decades, there are still details relating to cancer initiation, development, metastasis, and recurrence that have yet to be elucidated. A significant challenge to acquiring a comprehensive understanding is that multiple factors are in play in tumor microenvironments. Understanding the role of the host's microbiota has been overlooked in the past but has recently become of increasing interest [33, 34]. According to the findings of Vétizou et al., specific species of Bacteroides exert an effect upon the antitumor capacity of immune checkpoint inhibitors [35]. The effectiveness of antitumor medicine is reduced in germ-free mice with sterilized gut microbiota compared to SPF mice; when particular Bacteroides species were implanted into the intestines of mice, effectiveness was restored. Sivan et al. found that using immune checkpoint inhibitors in conjunction with a transplant of gut microbiota could significantly improve their antitumor efficacy [36].

Many symbiotic bacteria, such as *Lactobacillus* and *Lachnospiraceae*, are found in the intestines. These important species help with host digestion, maintain the intestinal environment by preventing intestinal colonization by oral bacterial clades, and exert anti-inflammatory effects. The importance of these bacteria is highlighted by studies that show microbiota dysbiosis can occur when these beneficial bacteria are absent, contributing to diabetes, inflammatory bowel disease, intestinal cancers, and obesity and adversely affecting the immune system [28, 37]. Insights into the development, metastasis, and recurrence of CRC can be increased by detailing features of dysbiosis in intestinal microbiota, especially of the large intestine. By developing a better understanding of host-microbiota interactions in CRC, the treatment of CRC patients can become more targeted. The tumor location in the intestines influences the severity of CRC [38] and the level of resistance to chemotherapy [39] in different patients. More than any other factors, diet and lifestyle are regarded as determining the probability of developing CRC. Ingesting polyphenols, such as apigenin, benefits gut microbiota and helps to protect against CRC [40, 41]. We explored the antitumor relationship between gut microbiota and apigenin through fecal bacteria transplantation experiments. Transplanting feces from apigenin donor mice into FMT-AP mice inhibited tumor carcinogenesis in the recipient mice.

As this study has shown, *in vivo* tumor carcinogenesis is inhibited by apigenin by influencing the gut microbiota. However, the precise mechanism by which apigenin affects the gut microbiota and exerts its antitumor effects have yet to be determined.

## Data Availability

The datasets used and/or analyzed during the current study are available from the corresponding author on reasonable request.
